# About Love

**Published:** 1881-03

**Authors:** 


### ABOUT LOVE.

[From the Boston Transcript.]

Mr. Factandfancy has noticed—

That the boy who is most afraid of the girls is the first to be corralled into matrimony.

That the little boys prefer boys to girls.

That they soon change, never to go back to their early love.

That the little girls love the girls best.

That they don't get over their preference so soon as the boys do—some of them never.

That women love the men because they love everything they have to take care of.

That men love women because they can't help it.

That the wife loves her husband so well that she has no thoughts for other men.

That the husband so loves his wife that he loves all women for her sake.

That the married man is apt to think himself all killing among the fair sex simply because he has found one woman fool enough to marry him.

That homely husbands are the best. They never forget the compliment paid them by their wives in accepting them.

That homely wives are the truest. They know how to make the most of what they have.

That the man who marries late in life does well.

That the man who marries young does better.

That the man who never marries is to be pitied.

That the woman who marries does well.

That the woman who does not marry does better nine times out of ten.

### HOAXING A MANAGER.

Mr. Henry C. Jarrett tells the following story : One evening, while the party were playing at the Opera-house in Detroit, a small boy approached him and holding out his hand, exhibiting fifteen cents, said :

"Please, mister, I would so much like to see 'Cinderella,' but that's all the money I've got."

The boy's manner touched Jarrett's tender spot, and after asking him two or three times if that was all the money he had, and receiving each time a pitiful affirmative answer, he gave him a quarter. The boy's countenance beamed with delight, and he did not know how to express his gratitude. Finally moving toward the street, he said :

"You don't know how thankful I am, sir. I am ever so much obliged to you, sir ; but now that you have been so generous, I guess I'll go to the other theatre and see 'Jack Sheppard.'"

